# Neutrophil-to-lymphocyte ratio in females as a predictor of embryo acquisition outcomes after IVF/ICSI treatment: a retrospective study

**DOI:** 10.3389/fendo.2025.1705636

**Published:** 2026-01-13

**Authors:** Liwen Shen, Li Chen, Lifen Chen, Huiping Jiang, Huifeng Gu, Liqun Lu

**Affiliations:** 1 Department of Center for Reproductive Medicine, Huzhou Maternity & Child Health Care Hospital, Huzhou, Zhejiang, China; 2 Nursing Department, Huzhou Maternity & Child Health Care Hospital, Huzhou, Zhejiang, China

**Keywords:** female, fertilization *in vitro*, infertility, lymphocytes, neutrophils

## Abstract

**Objective:**

We aimed to analyze the correlation between the neutrophil-to-lymphocyte ratio (NLR), and the treatment outcomes for infertile women after *in vitro* fertilization (IVF) using embryo transfer technology.

**Methods:**

This retrospective study enrolled women with infertility at Huzhou Maternal and Child Health Care Hospital who underwent *in vitro* IVF procedures. Patient data were collected from a reproductive electronic medical record system. We divided 976 participants into positive and negative groups based on embryo availability after IVF. Age, education level, body mass index, infertility type, etiology, miscarriage history, ovarian stimulation protocols, baseline follicle stimulating hormone levels, anti-Müllerian hormone and NLR were compared in both groups. We investigated the association between NLR and IVF outcomes using logistic regression analysis with multi-model adjustments. The receiver operating characteristic (ROC) curve was used to evaluate the screening efficacy of NLR, The subgroup analysis revealed risk variations among different groups. Finally, we performed sensitivity analysis by modifying control values and conducting logistic regression using NLR quartiles.

**Results:**

Multimodal adjusted logistic regression analysis revealed a significant association between the second quartile of NLR and negative outcomes of IVF treatment, with an OR value and 95% confidence interval of 0.28 (0.10-0.67). The area under the ROC curve was 0.850 <. We observed an interaction between NLR quartiles and infertility types, particularly positive correlations between primary infertility, female-specific infertility factors, and no history of miscarriage with IVF outcomes at the second quartile. When the second quartile was adjusted as a reference value, the three remaining quartiles exhibited statistically significant differences compared to the second quartile (*p* for trend = 0.045).

**Conclusion:**

We recommended dynamically monitoring NLR during the cycle of ovulation induction and advocating individualized inflammatory management based on the cause of infertility to ensure the effectiveness of IVF treatment and prevent resource wastage.

## 1 Introduction

Infertility has become an increasingly serious public health problem worldwide. Epidemiological surveys indicate that 8-12% of couples are affected, and the incidence continues to rise each year (1). Although *in vitro* fertilization (IVF)-embryo transfer and related technologies have successfully helped many women achieve pregnancy, the overall success rate remains suboptimal. The uncertainty of treatment outcomes imposes a negative impact comparable to the substantial economic, physical and psychological burdens associated with infertility. Therefore, there is an urgent need for reliable predictive tools to assess treatment outcomes.

Blood counts such as absolute eosinophil count, absolute neutrophil count, absolute lymphocyte count and absolute monocyte count can be performed to monitor risk factors associated with the occurrence of immunization-related adverse events (2, 3). The neutrophil-to-lymphocyte ratio (NLR), a related derived indicator, has demonstrated significant value in the prediction of disease outcomes. It is an emerging systemic inflammation marker, is calculated based on routine blood count, offering advantages of low cost, rapidity, and convenience. It has been confirmed as a robust prognostic factor for infectious diseases (such as sepsis, pneumonia and COVID-19) and cancer. The changes in NLR concentrations can effectively reflect the state of inflammation and immune loss of balance (4). In addition, NLR can also reflect the immune status in the absence of infectious diseases.

Recent studies have found that peripheral biomarkers like female androgen levels and the progesterone-to-mature-oocytes index positively predict assisted reproductive technology outcomes (5–7). In recent years, chronic inflammation has garnered increasing attention in the diagnosis and treatment of female infertility, as it affects both natural conception and the therapeutic outcomes of assisted reproductive technologies (8). Furthermore, evidence indicates that NLR is associated with various female reproductive conditions, such as endometriosis, breast cancer (9, 10). Therefore, this study attempts to evaluate the role of the NLR in predicting outcomes of assisted reproduction, with the aim of providing a simple and cost-effective tool for this purpose.

## 2 Materials and methods

### 2.1 Study design and population

This was a single-center retrospective case-control clinical study conducted at Huzhou Maternal and Child Health Care Hospital. We collected and analyzed data from patients who underwent *in vitro* IVF treatment between January 1, 2023 and December 31, 2024. Patient data were retrieved from the assisted reproduction management system. All patients were diagnosed with infertility and confirmed to meet the criteria for IVF treatment based on their detailed medical history, and clinical evaluation. Exclusion criteria were repeated IVF treatments, cycle cancellations, systemic diseases causing inflammation, autoimmune diseases, a history of cancer, and the use of medications other than those for *in vitro* IVF treatment. This study was conducted in accordance with the Declaration of Helsinki and was approved by the Review Committee of Huzhou Maternal and Child Health Care Hospital. Considering this was a retrospective study, the ethics committee waived the requirement for written informed consent.

### 2.2 Definition of NLR and IVF outcome

Whole blood was collected from patients before the anticipated trigger (when the dominant follicle was ≥14 mm). A flow-cytometry-based SYSMEX automated hematology analyzer was used to quantify neutrophils and lymphocytes in the blood for determining NLR, The calculation formula of NLR is shown as follows.


$$NLR=\frac{A\text{b}solute\cdot Neutrophil\cdot Count(10^{9}/L)}{Absolute\cdot Lymphocyte\cdot Count(10^{9}/L)}$$

Based on the outcomes from the initiation of IVF treatment (including Intracytoplasmic Sperm Injection, ICSI) to either embryo transfer or embryo cryopreservation, treatment results were categorized into two groups: positive and negative. The positive group referred to patients who obtained transferable embryos after IVF treatment, and the outcomes included fresh embryo transfer or complete embryo cryopreservation without transfer. The negative group referred to patients who did not obtain transferable embryos after IVF treatment, and the outcomes included no oocyte retrieval, failed fertilization, abnormal fertilization, or failure to culture transferable embryos.

### 2.3 Assessment of covariates

A multivariable adjustment model was constructed using variables such as age, education level, BMI, type of infertility, cause of infertility, number of miscarriages, ovarian stimulation protocol, baseline FSH level, and AMH level. These variables were adjusted to reduce the confounding effect between NLR and IVF outcomes. Education level was categorized into three groups based on whether participants had completed high school, or below college, or above college. Infertility type was classified as primary or secondary infertility based on whether the patient had a history of pregnancy. the number of miscarriages was categorized as 0, 1–2 times, and 3 times or more based on the miscarriage history of the patient. Ovarian stimulation protocols were classified into follicular phase long protocol, luteal phase long protocol, antagonist protocol, mild stimulation protocol, and natural cycle. The embryo grading methods are detailed in previously published literature by the research team (11).

### 2.4 Statistical analysis

In the descriptive analysis, continuous variables were not normally distributed and were presented as medians with 5th and 95th percentiles. Differences between groups were determined using the Wilcoxon test. Categorical variables were presented as percentages, and intergroup differences were evaluated using the chi-square test. NLR was analyzed both as a continuous variable and by quartiles.

Multivariate logistic regression analysis was performed to investigate the association between NLR and IVF treatment outcomes. Three models were developed to calculate odds ratios (OR) and 95% confidence intervals (CI). Model 1 included no covariate adjustments. Model 2 was adjusted for age, BMI, and educational level. Model 3 was further adjusted for infertility type, cause of infertility, number of miscarriages, ovarian stimulation protocol, baseline FSH level, and AMH level in addition to the variables in Model 2. The receiver operating characteristic (ROC) curve and the area under the curve (AUC) were used to evaluate the predictive value of NLR for IVF treatment outcomes. Subgroup analyses and interaction tests were performed to assess potential interactions between NLR and covariates. Finally, regression analyses based on the three models were repeated using the second quartile of NLR as the reference category to evaluate the robustness of the study findings.

All statistical analyses were performed using Decision Linnc 1.0 (12). A two-sided p-value < 0.05 was considered statistically significant.

## 3 Results

### 3.1 Study population

**Figure 1:**
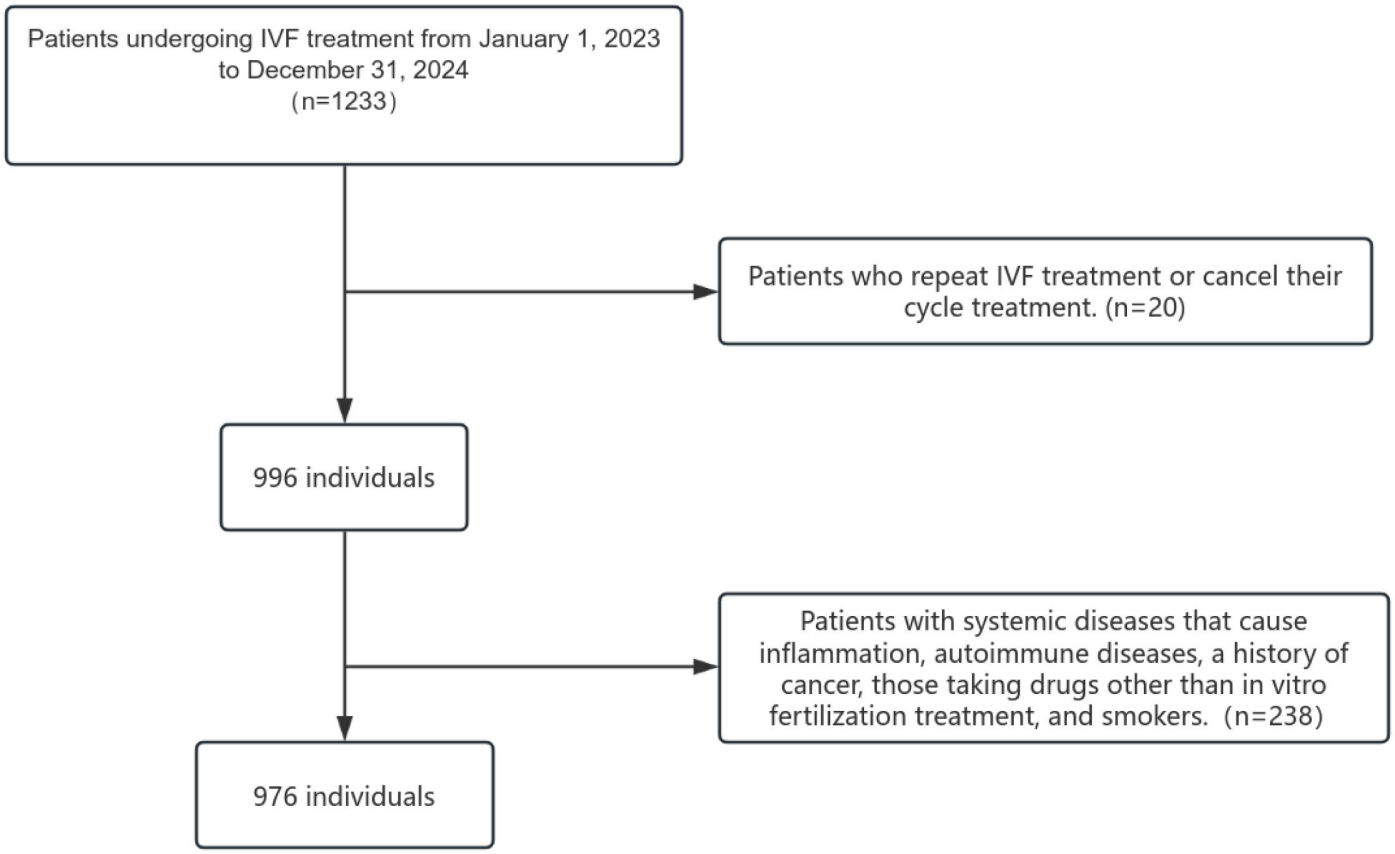
Flow diagram of the patients selection process.

The patient selection for the study is summarized in the flow chart (**Figure 1**). We included 976 patients in this study, 901 obtaining transferable embryos after IVF and 75 had poor outcomes. There was a significant difference in age between the two groups, and the negative group exhibiting poor IVF treatment outcomes was significantly older than the positive group (*p* < 0.001). A significant difference was also observed in the number of miscarriages between the two groups (*P* = 0.005). Basal FSH levels were significantly higher in the negative outcome group compared to those in the positive group (*p* < 0.001), In contrast, AMH levels were significantly lower in the negative group than in the positive group (*p* < 0.001). When NLR was analyzed as a continuous variable, no significant difference was found between the two groups. However, a significant difference in distribution was observed when NLR was categorized into quartiles (*p* = 0.030). See **Table 1** for details.

**Table 1:** Comparison of characteristics of participants with different IVF outcomes.

Variable	Overall (n = 976)	Positive (n = 901)	Negative (n = 75)	*P* Value
Age(year)	31.0 (28.0 - 35.0)	31.0 (28.0 - 35.0)	36.0 (30.0 - 41.0)	<0.001
Education level,n(%)				0.268
≤Hight school	504 (51.6%)	464 (51.5%)	40 (53.3%)	
College	439 (45.0%)	409 (45.4%)	30 (40.0%)	
≥Master	22 (2.3%)	18 (2.0%)	4 (5.3%)	
not recorded	11 (1.13%)	10 (1.1%)	1 (1.3%)	
BMI(kg/m^2^)	22.4 (20.3 - 25.0)	22.4 (20.3 - 25.0)	22.1 (20.3 - 24.7)	0.796
Infertility type, n (%)				0.522
Primary infertility	451 (46.2%)	419 (46.5%)	32 (42.7%)	
Secondary infertility	525 (53.8%)	482 (53.5%)	43 (57.3%)	
Infertility causes,n (%)				0.309
Female factor	798 (81.8%)	732 (81.2%)	66 (88.0%)	
Male factor	79(8.1%)	75 (8.3%)	4 (5.3%)	
Couple factor	70 (7.2%)	65 (7.2%)	5 (6.7%)	
Unexplained infertility	29(3.0%)	29 (3.2%)	0 (0%)	
Number of abortions, n (%)				0.005
0	642(65.8%)	597 (66.3%)	45 (60.0%)	
1-2	297 (30.4%)	275 (30.5%)	22 (29.3%)	
>2	37 (3.8%)	29 (3.2%)	8 (10.7%)	
Ovarian stimulation protocol, n (%)				0.888
Follicular phase GnRH-a Long protocol	83 (8.5%)	76 (8.4%)	7 (9.3%)	
Luteal phase GnRH-a Long protocol	442 (45.3%)	408 (45.3%)	34 (45.3%)	
Antagonist protocol	244 (25.0%)	228 (25.3%)	16 (21.3%)	
Microstimulation protocol	181 (18.6%)	166 (18.4%)	15 (20.0%)	
Natural cycle protocol	26 (2.7%)	23 (2.6%)	3 (4.0%)	
Basal FSH (mIU/ml)	6.6 (5.6 - 7.6)	6.5 (5.5 - 7.5)	8.60 (6.8 - 11.6)	<0.001
AMH (ng/ml)	3.3 (1.7 - 5.3)	3.5 (1.9 - 5.5)	0.94 (0.4 - 1.9)	<0.001
NLR	2.0 (1.5 - 2.7)	2.0 (1.5 - 2.7)	2.08 (1.5 - 2.8)	0.482
NLR quartile,n (%)				0.030
Q1	244 (25.0%)	221 (24.5%)	23 (30.7%)	
Q2	244 (25.0%)	236 (26.2%)	8 (10.7%)	
Q3	244 (25.0%)	222 (24.6%)	22 (29.3%)	
Q4	244 (25.0%)	222 (24.6%)	22 (29.3%)	

n, number; BMI, body mass index; AMH, anti-Mullerian hormone; NLR, neutrophil to lymphocyte ratio.

### 3.2 Multi-model logistic regression analysis

When NLR was analyzed as a continuous variable, no significant association with IVF treatment outcomes was found in any of the three models. However, when NLR was divided into quartiles, a significant association was observed between the second quartile and negative IVF outcomes across all three models (Model 1: OR = 0.33, *P* = 0.008; Model 2: OR = 0.31, *P* = 0.006; Model 3: OR = 0.28, *P* = 0.007). See **Table 2** for details.

**Table 2:** Exploring the associations between NLR and IVF outcomes logistic regression in different models.

Variables	Model 1		Model 2		Model 3	
	OR (95%*CI*)	*p* Value	OR (95%*CI*)	*p* Value	OR (95%*CI*)	*p* Value
NLR (continuous)	1.08 (0.91-1.24)	0.325	1.08 (0.90-1.26)	0.346	1.12 (0.91-1.35)	0.262
NLR (quartile)						
Q1	Reference					
Q2	0.33 (0.13-0.72)	0.008	0.31 (0.12-0.69)	0.006	0.28 (0.10-0.67)	0.007
Q3	0.95 (0.51-1.76)	0.876	0.82 (0.43-1.56)	0.544	0.84 (0.42-1.69)	0.627
Q4	0.95 (0.51-1.76)	0.876	0.82 (0.43-1.56)	0.541	0.91 (0.44-1.84)	0.786

Model 1: Non-adjusted; Model 2: Adjusted for age, education level and BMI; Model 3: Further adjusted for infertility type, infertility causes, number of abortions, ovarian stimulation protocol, basal FSH and AMH; OR, Odds Ratio; NLR, Neutrophil to lymphocyte ratio.

### 3.3 ROC curve analysis

**Figure 2:**
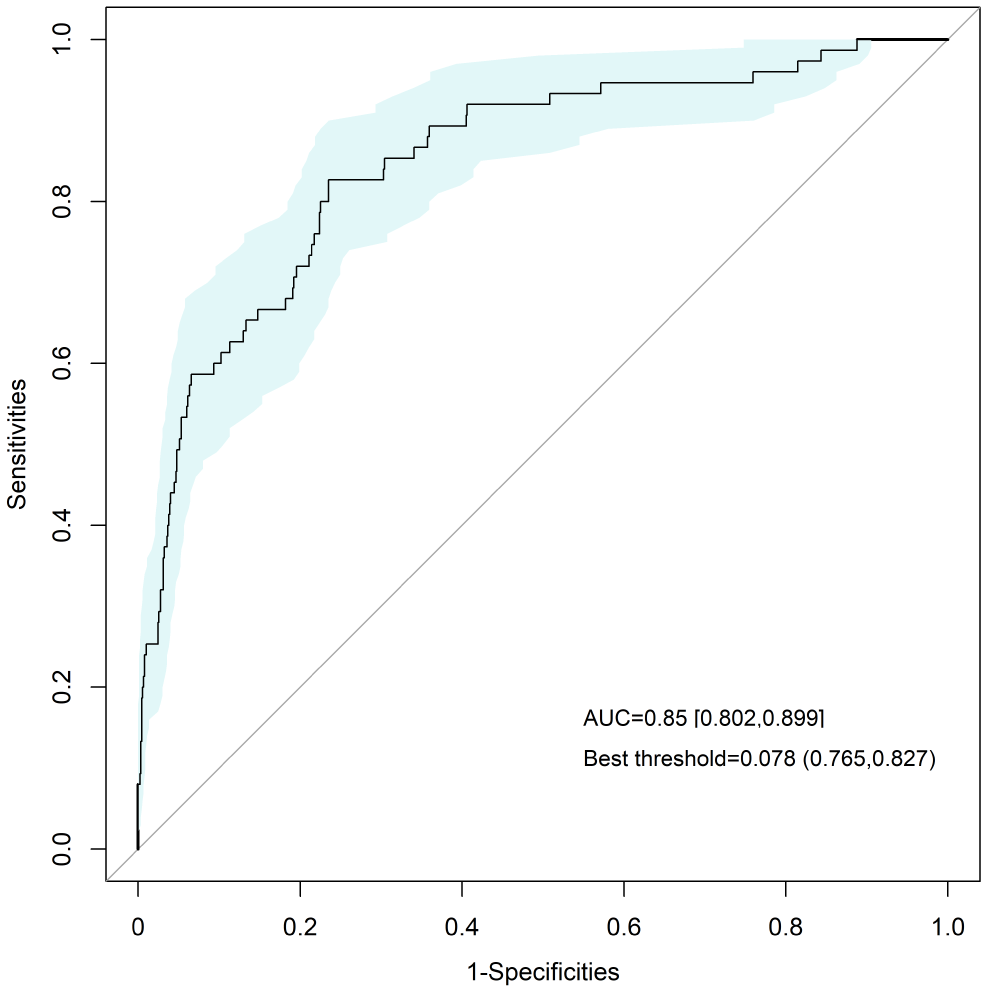
ROC curve analysis of NLR for predicting embryo acquisition outcomes.

The AUC was 0.850(95%*CI*: 0.802–0.899, *p* < 0.001), with a sensitivity of 0.827 and a specificity of 0.765. The theoretically optimal truncation value is 0.078, corresponding to a Youden index of 0.591. See **Figure 2** for details.

### 3.4 Subgroup analysis and interaction

**Figure 3:**
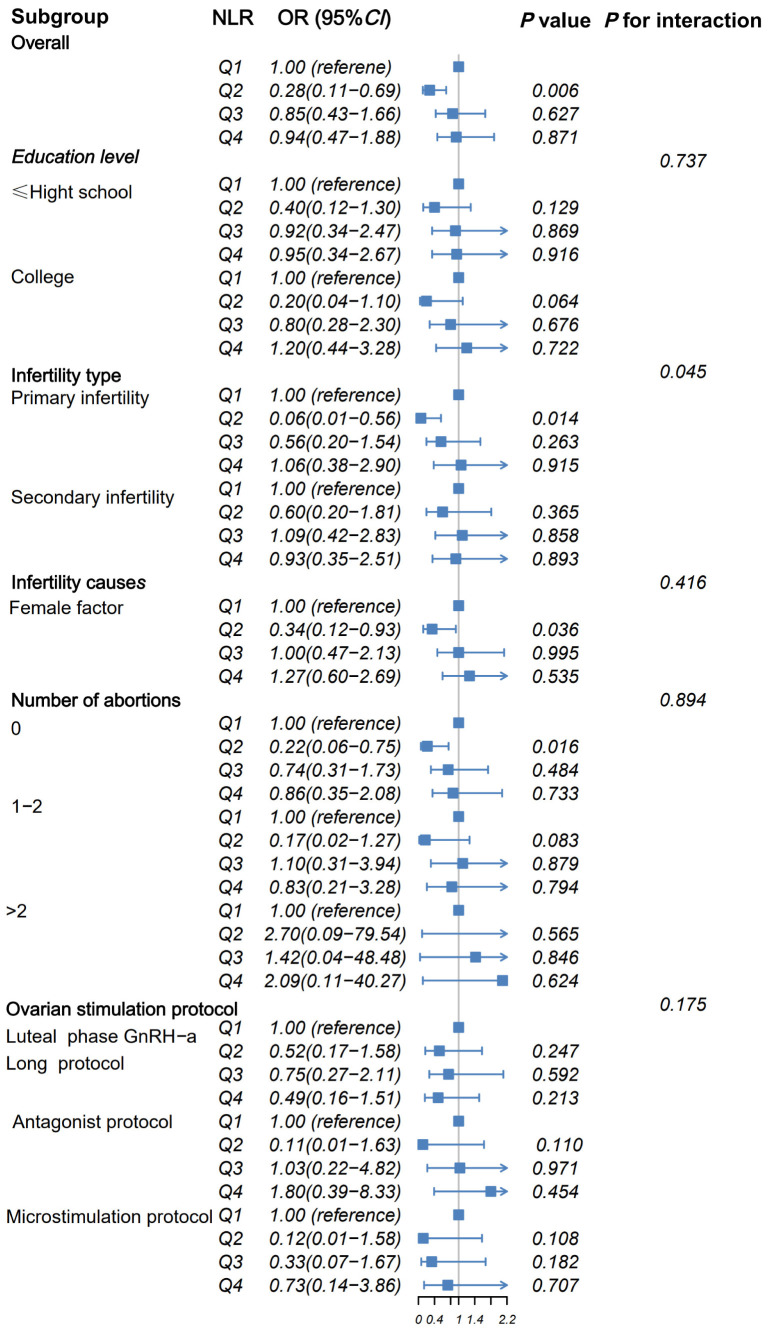
Subgroup analysis of NLR and embryo acquisition outcomes.

After subgroup analysis and interaction analysis, we found that there was an interaction between the quartile of NLR and the type of infertility, and the second quartile of primary infertility, female factor infertility, and no history of abortion were protective factors for IVF outcomes (see **Figure 3**). Based on the logistic regression analysis, significant differences in IVF treatment outcomes were observed for the second quartile of NLR. Therefore, the second quartile was set as the reference group. Compared to this reference, all other three quartiles showed significant differences in IVF treatment outcomes, and a statistically significant trend was observed (*p* for trend = 0.045). (see **Table 3**)

**Table 3:** Exploring the associations between NLR (quartile) and IVF outcomes logistic regression in different models.

Variables	Model 1		Model 2		Model 3	
	OR (95%*CI*)	*p* Value	OR (95%*CI*)	*p* Value	OR (95%*CI*)	*p* value
NLR (quartile)						
Q2	Reference					
Q1	3.07 (1.40-7.45)	0.008	3.27 (1.46-8.06)	0.006	3.62 (1.49-9.81)	0.007
Q3	2.92 (1.33-7.12)	0.011	2.68 (1.19-6.64)	0.023	3.04 (1.26-8.23)	0.019
Q4	2.92 (1.33-7.12)	0.011	2.68 (1.19-6.63)	0.023	3.28 (1.32-9.04)	0.014
*P* for trend		0.029		0.079		0.045

Model 1: Non-adjusted; Model 2: Adjusted for age, education level and BMI; Model 3: Further adjusted for infertility type, infertility causes, number of abortions, ovarian stimulation protocol, basal FSH and AMH; OR: Odds Ratio; NLR: Neutrophil to lymphocyte ratio.

## 4 Discussion

This is the first study to examine the relationship between the NLR, an inflammatory marker, and the likelihood of obtaining transferable embryos using IVF techniques. The acquisition of transferable embryos is a necessary prerequisite for the final step of embryo transfer in IVF. Determining whether transferable embryos can be obtained through IVF enables earlier prediction of treatment outcomes, which may help avoid unnecessary use of medical resources. Using regression analysis across multiple models, we found that NLR in the second quartile (1.53-1.95) effectively predicts favorable treatment outcomes after IVF, with relatively high predictive accuracy. Furthermore, among women with NLR values within this range, those with characteristics such as primary infertility, female-factor infertility, and no history of miscarriage were more likely to achieve favorable treatment outcomes. An interaction was observed between infertility type and NLR.

Most studies focus on pregnancy outcomes following IVF treatment (13), and current research on the association between NLR and outcomes of assisted reproductive treatment is limited. Siristatidis C et al. found a correlation between the highest-quartile (Q4) NLR and the success rate of assisted reproductive treatment, however, further logistic regression analysis did not confirm the independent predictive value of the highest-quartile NLR. In addition, the sample size was too small, and further studies with larger sample sizes are needed to verify the predictive efficacy of NLR (14). We observed a positive correlation between the second quartile of NLR and the embryo acquisition rate in IVF procedures, suggesting that the second quartile of NLR may represent the “optimal inflammatory window”. Although the optimal NLR cut-off value of 0.078, determined by ROC analysis, falls below its actual measurable range. This phenomenon is primarily attributable to the class imbalance within the dataset (with positive outcomes being predominant) and the presence of individuals with high NLR values in the positive group. Nonetheless, an AUC of 0.850 demonstrates that NLR retains significant discriminatory potential. Therefore, we conclude that while NLR is not suitable as a standalone diagnostic tool, it holds important value as a reference indicator for risk stratification of IVF treatment outcomes, and It can significantly complement previous studies predicting IVF outcomes using hormones, ovarian reserve, or composite indices.

Embryo acquisition results from the combination and normal dissociation of sperm and egg, and a decline in the number of primordial follicles due to various causes leads to infertility and treatment failure in assisted reproductive therapy (15). The formation of follicles and the maturation of oocytes depend on the ovarian microenvironment (16). The inflammatory process plays a critical role in regulating folliculogenesis and ovarian remodeling (17). A suppression of ovarian hormones during IVF, can increase oxidative stress levels (18), thereby promoting chronic inflammation. Therefore, we hypothesize that moderate inflammation induced by IVF may contribute to favorable outcomes of IVF treatment. A retrospective study on female patients undergoing embryo implantation using IVF techniques found that a low quartile NLR could effectively predict IVF outcomes (clinical pregnancy rate and viable pregnancies) (19), further supporting this hypothesis.

NLR reflects the systemic inflammatory state. An increase in neutrophil count and a reduction in lymphocyte count may lead to increased NLR levels. A moderate inflammatory reaction can benefit the immune system by promoting apoptosis and clearance of inflammatory cells, whereas excessive inflammation may result in tissue damage (20, 21). Neutrophils are frontline cells of the innate immune system. They possess antimicrobial mechanisms, and the recruitment of neutrophils in the early stages of inflammation is beneficial for combating microbes, regulating the severity of infection, and restoring internal balance. However, if neutrophils remain active after their intended functions, they may cause damage to the host (22). In addition, a significant increase in neutrophils count indicates that the body is experiencing an inflammatory attack. During this process, cytokines, such as interleukin-1 (IL-1) and interleukin-6 (IL-6) are released. These cytokines disrupt endocrine homeostasis and the ovarian microenvironment, thereby impairing oocyte quality, fertilization potential, and embryo development (23–26). Moreover, neutrophils can suppress the proliferation of lymphocytes and induce lymphocyte apoptosis (27, 28). In the sensitivity analysis of this study, it was further confirmed that, compared with the second quartile of NLR, higher quartiles of NLR were associated with failure to obtain embryos after IVF treatment. Previous studies have similarly shown that excessive inflammation negatively affects IVF outcomes (29).

Immune cells induced by pregnancy establish maternal immune tolerance, which subsequently leads to immune memory (30, 31). Some researchers suggest that localized inflammatory states in women and subsequent immune dysregulation may contribute to adverse outcomes in assisted reproductive treatments (32, 33). We found a significant interaction between NLR and types of infertility, therefore, we speculate that in patients with a history of pregnancy, the predictive efficacy of NLR is weakened, whereas in primary infertility, in which the female has not been exposed to pregnancy-related immune regulation, the predictive efficacy of NLR has high sensitivity. Low-grade chronic inflammation is commonly associated with several gynecological diseases related to female infertility, such as salpingitis, endometriosis, and endocrine disorders (34). Therefore, low inflammatory markers may be more predictive in women with infertility. During normal pregnancy, the maternal immune system balances physiological changes in the mother and fetal growth (35). Dimitriadis E et al. suggested that excessive inflammation is associated with recurrent spontaneous abortion (36). Here, we found that patients without a history of miscarriage were more likely to achieve positive treatment outcomes compared to those with a history of miscarriage, possibly indicating a more stable immune status in the group without miscarriage history.

This study has certain limitations. First, as a retrospective study, it cannot directly establish a causal relationship between NLR and embryo acquisition in IVF, and further prospective studies are needed to explore the mechanism by which NLR affects the embryo acquisition rate in IVF. Second, this approach, along with the limited number of negative outcomes and simple etiological classification, may affect the statistical power. Future studies with larger sample sizes and more refined etiological categorizations of female are needed to enhance the generalizability of the findings. And last, embryo acquisition is jointly influenced by both male and female factors, however, this study only investigated the impact of NLR levels in females, it is necessary to further collect male-related factors to reduce bias in the study conclusions.

## 5 Conclusion

We identified a correlation between low percentile NLR and IVF outcomes, suggesting dynamic monitoring of NLR during ovarian stimulation cycles and anti-inflammatory pretreatment for patients with primary infertility and high NLR. In patients with primary infertility, female factors, and no history of miscarriage indicated better fertility potential. Individualized inflammation management based on the etiology of infertility is advocated to ensure the effectiveness of IVF treatment and avoid wastage of resources.

## Data Availability

The raw data supporting the conclusions of this article will be made available by the authors, without undue reservation.
